# Pre-operative anaemia is associated with total morbidity burden on days 3 and 5 after cardiac surgery: a cohort study

**DOI:** 10.1186/s13741-017-0057-4

**Published:** 2017-01-21

**Authors:** Julie Sanders, Jackie A. Cooper, Daniel Farrar, Simon Braithwaite, Updeshbir Sandhu, Michael G. Mythen, Hugh E. Montgomery

**Affiliations:** 1 St Bartholomew’s Hospital, Barts Health NHS Trust, London, UK; 2 Centre for Cardiovascular Genetics, University College London, London, UK; 3 Department of Cardiac Anaesthesia and Critical Care, University College London Hospitals NHS Foundation Trust, London, UK; 4 UCL Medical School, University College London, London, UK; 5 University College London Hospitals NHS Trust, London, UK; 6 Institute for Sport, Exercise and Health, University College London, 1st Floor 170 Tottenham Court Rd, London, W1T 7HA UK

**Keywords:** Anaemia, Post-operative morbidity, Total morbidity burden, Red blood cell transfusion, Cardiac surgery

## Abstract

**Background:**

Pre-operative anaemia is associated with mortality and red blood cell (RBC) transfusion requirement after cardiac surgery. However, the effect on post-operative total morbidity burden (TMB) is unknown. We explored the effect of pre-operative anaemia on post-operative TMB.

**Methods:**

Data were drawn from the Cardiac Post-Operative Morbidity Score (C-POMS) development study (*n* = 442). C-POMS describes and quantifies (0–13) TMB after cardiac surgery by noting the presence/absence of 13 morbidity domains on days 3 (D3), 5 (D5), 8 (D8) and 15 (D15). Anaemia was defined as a haemoglobin concentration below 130 g/l for men and 120 g/l for women.

**Results:**

Most patients were White British (86.1%) and male (79.2%) and underwent coronary artery bypass surgery (67.4%). Participants with pre-operative anaemia (*n* = 137, 31.5%) were over three times more likely to receive RBC transfusion (OR 3.08, 95%CI 1.88–5.06, *p* < 0.001), had greater D3 and D5 TMB (5 vs 3, *p* < 0.0001; 3 vs 2, *p* < 0.0001, respectively) and remained in hospital 2 days longer (8 vs 6 days, *p* < 0.0001) than non-anaemic patients. Transfused patients remained in hospital 5 days longer than non-transfused patients (*p* < 0.0001), had higher TMB on all days (all *p* < 0.001) and suffered greater pulmonary, renal, GI, neurological, endocrine and ambulation morbidities (*p* 0.026 to <0.001). Pre-operative anaemia and RBC transfusion were independently associated with increased C-POMS score.

**Conclusions:**

Pre-operative anaemia and RBC transfusion are independently associated with increased post-operative TMB. Understanding TMB may assist in post-operative patient management to reduce morbidity. We recommend the use of the C-POMS tool as a standard outcome tool in further studies.

## Background

Anaemia, defined as circulating haemoglobin (Hb) concentration level below 130 g/l for men and 120 g/l for women (World Health Organization 2008), affects 24.8% of the global population (World Health Organization 2008), and up to 54.4% of cardiac surgery patients (Hung et al. 2011) are anaemic prior to surgery.

Since Hb is the circulation’s oxygen-carrying molecule, anaemia is associated with decreased blood oxygen content. Unless compensated for by increased blood flow, inadequate tissue oxygen delivery (Kurtz et al. 2010) may impair organ function. Furthermore, iron is not only essential for the synthesis of Hb’s haem moiety but also plays an important role in oxidative metabolism (Dunn et al. 2007). Iron deficiency may thus directly impair mitochondrial oxidative metabolism and adenosine triphosphate (ATP) synthesis through direct mitochondrial effects, as well as through anaemia and resulting impairment of oxygen delivery (Davies et al. 1982). In pre-operative anaemic patients, these deficits follow the patient into surgery, which itself is associated with a substantial and sustained increase in metabolic activity and hence in oxygen demand (Vallet and Futier 2010). By limiting the capacity to respond to this increased metabolic demand, pre-operative anaemia might thus be postulated to impair post-operative recovery.

Indeed, pre-operative anaemia has been associated with adverse outcome after cardiac surgery and has been associated with higher mortality (Hung et al. 2011; Zindrou et al. 2002; Cladellas et al.; van Straten et al. 2009; De Santo et al. 2009; Boening et al. 2011; Miceli et al. 2014), longer stay on the intensive care unit (ICU) (Hung et al. 2011; De Santo et al. 2009) and in hospital (Cladellas et al.; De Santo et al. 2009; Miceli et al. 2014; Kulier et al. 2007) and a higher incidence red blood cell (RBC) transfusion (De Santo et al. 2009; Boening et al. 2011). However, the evidence relating to the influence of pre-operative anaemia on post-operative morbidity is divided and only relates to specific outcomes, for example stroke (Cladellas et al.; Miceli et al. 2014; Fowler et al.) and renal dysfunction (Cladellas et al.; Miceli et al. 2014; Fowler et al.; Carrascal et al. 2010). Thus, whether anaemia is an independent risk factor for general morbidity after cardiac surgery (Fowler et al.) and the scale of this impact on total morbidity burden (TMB) has yet to be reported.

Thus, we explored the association between pre-operative anaemia and RBC transfusion requirement with TMB after cardiac surgery.

## Methods

### Participants

Patients were drawn from the Cardiac Post-Operative Morbidity Score (C-POMS) development and validation study; the methods describing how the C-POMS measurement tool was developed and validated are detailed elsewhere (Sanders et al.). In brief, patients undergoing any form of adult cardiac surgery (excluding cardiac surgery for a congenital heart condition or a cardiomyopathy) between January 2005 and November 2007 at the Heart Hospital, University College London Hospitals NHS Trust, UK, and who gave written informed consent were eligible for inclusion. Excluded were those <18 years old, undergoing emergency surgery, who were enrolled in clinical intervention trials or who died within 5 days of surgery.

### Defining anaemia

Anaemia was defined as a haemoglobin (Hb) concentration below 130 g/l for men and 120g/l for women (Organisation WH 2008).

### Outcome measurements

Post-operative morbidity and hence total morbidity burden were defined using the C-POMS tool (Table 1).

**Table 1:** The Cardiac Post-Operative Morbidity Score (C-POMS) (Sanders et al.)

Morbidity type	C-POMS criteria
Pulmonary	Presence of one or more of the following: ■ New requirement for oxygen or respiratory support (including nebuliser therapy or request for chest physiotherapy on or after D5) ■ Pleural effusion requiring drainage
Infectious	Presence of one or more of the following: ■ Currently on antibiotics ■ A temperature of >38 °C in the last 24 h ■ A white cell count/CRP level requiring in-hospital review or treatment
Renal	Presence of one or more of the following: ■ Decreased urine output requiring intervention (including IV furosemide) ■ Increased serum creatinine (>30% from pre-operative level) ■ Urinary catheter in situ ■ New urinary incontinence ■ Serum potassium abnormalities requiring treatment
Gastrointestinal	Presence of one or more of the following: ■ Unable to tolerate an enteral diet for any reason including nausea, vomiting and abdominal distension ■ The presence of a nasogastric tube ■ Diagnosis of a gastrointestinal bleed ■ Diarrhoea
Cardiovascular	Presence of one or more of the following: ■ The use of inotropic therapy for any cardiovascular cause ■ Pacing wires (on or after D5) and/or requiring temporary or new permanent pacing ■ Diagnostic tests or therapy within the last 24 h for any of the following: (1) new MI or ischaemia, (2) hypotension (requiring fluid therapy, pharmacological therapy or omission of pharmacological therapy), (3) atrial or ventricular arrhythmias, (4) cardiogenic pulmonary oedema, thrombotic event (requiring anticoagulation), (5) hypertension (pharmacological therapy or omission of pharmacological therapy)
Neurological	New neurological deficit (including confusion, delirium, coma, lack of coordination, drowsy/slow to wake, poor swallow, blurred vision, sedated, changing loss of consciousness)
Haematological	Presence of one or more of the following: ■ Untherapeutic INR requiring pharmacological therapy or omission of pharmacological therapy ■ Requirement for any of the following within the last 24 h: packed erythrocytes, platelets, fresh-frozen plasma or cryoprecipitate
Wound	Presence of one or more of the following: ■ Wound dehiscence requiring surgical exploration or drainage of pus from the operation wound with or without isolation of organisms ■ Chest drains ■ Wound pain significant enough to require continuing or escalating analgesic intervention
Pain	Post-operative pain significant enough to require parenteral opioids and/or continuing or additional analgesia.
Endocrine	New or additional requirements for blood sugar management
Electrolyte	Electrolyte (including sodium, urea, phosphate) imbalance requiring oral or intravenous intervention (not including potassium as included in renal category)
Review	Remaining in hospital for further review, investigation and/or procedure
Assisted ambulation	A new or escalated post-operative requirement for mobility assistance (including wheelchair, crutches, zimmer frame, walking sticks or assistance)
Non-C-POMS related reasons for delayed discharge on D5, D8 and D15 which the PDG decided should also be routine data collection in C-POMS on these days.	
Non-morbidity reason for delayed discharge	Where C-POMS is ‘0’ but the patient remains in hospital, state the reason for lack of discharge: *Social reasons; Equipment at home; Mobility* (ongoing physic and OT needs); *Institutional failure* (transport not booked, OPA or follow-up not arranged); *Delayed discharge* (lack of rehab or other bed); *Discharge planned for today; Other medical reason*

*CRP* C-reactive protein, *IV* intravenous, *MI* myocardial infarction, *INR* international normalised ratio, *OPA* out-patient appointment, *OT* occupational therapy

### RBC transfusion

Allogenic RBC transfusions were defined as any RBC transfusion given to the participant in the intra- and post-operative period prior to discharge from hospital and were collected by staff using the C-POMS tool (Table 1).

At the time of data collection, there was no uniform protocol for blood transfusion, although the unit operated a generally restrictive transfusion policy. Trust guidelines stipulated that RBC transfusion was strongly indicated when the haemoglobin was below 70 g/l. Since November 1999, all allogeneic blood components produced in the UK have been subjected to leucocyte depletion (LD) whereby ≥99% of units have <5 × 10^6^ leucocytes and >90% <1 × 10^6^ leucocytes (Service UKBT and T 2007).

### Total morbidity burden: C-POMS summary score

Post-operative morbidity was prospectively assessed on days 3 (D3), 5 (D5), 8 (D8) and 15 (D15) after cardiac surgery using the C-POMS tool (Sanders et al.). This represents TMB as a summary score (0–13), derived by noting the new or escalating presence or absence of 13 morbidity domains. Thus, the higher the score, the more morbidity experienced by the patient (Table 1).

### Post-operative length of stay

Post-operative length of stay (LOS) was defined as the number of days from surgery (day of operation day 0) to discharge from hospital. This included any days spent in a receiving hospital following transfer from the operative hospital.

### Other clinical data

Other clinical information including patient demographic details, relevant medical history, symptoms, risk factors, intra-operative details and general outcome variables (as shown in Table 2) were extracted from the C-POMS study. These were originally obtained from the medical and nursing records and the Society of Cardiothoracic Surgery of Great Britain and Ireland’s local database.

**Table 2:** Baseline characteristics (*n* = 442, unless otherwise stated). All values *n*(%) unless otherwise stated

	Overall	Anaemic	Not anaemic	*P* (anaemic vs not anaemic)
	(*n* = 442)	(*n* = 139)	(*n* = 303)	
	Frequency (%)/mean ± SD	Frequency (%)/mean ± SD	Frequency (%)/mean ± SD	
Demographics				
Age (mean/years)	66.5 ± 10.6	69.5 ± 10.5	65.11 ± 10.4	0.000
Female gender	92 (20.8)	32 (23.0)	60 (19.8)	0.451
Ethnicity (White British) (*n* = 438)	377 (86.1)	106 (76.8)	271 (90.3)	0.001
Medical history				
Renal (dialysis)	7 (1.6)	6 (4.3)	1 (0.3)	0.005
History of previous MI (*n* = 415)	147 (35.4)	49 (38.0)	98 (34.3)	0.375
Re-operation	18 (4.1)	8 (5.8)	10 (3.3)	0.299
Cerebrovascular accident	16 (3.6)	9 (6.5)	7 (2.3)	0.050
Gastrointestinal disease	50 (11.3)	23 (16.5)	2 (8.9)	0.023
Congestive heart failure (*n* = 415)	13 (3.1)	8 (6.2)	5 (1.7)	0.034
Symptoms				
NYHA class (*n* = 441)				
- I	114 (25.9)	32 (23.0)	82 (27.2)	0.245
- II	204 (46.3)	65 (46.8)	139 (46.0)	
- III	101 (22.9)	31 (22.3)	70 (23.2)	
- IV	22 (5.0)	11 (2.5)	11 (3.6)	
Cardiac risk factors				
Smoking				
- Current	49 (11.1)	14 (10.1)	35 (11.6)	0.891
- Ex	244 (55.2)	77 (55.4)	167 (55.1)	
- Never	149 (33.7)	48 (34.5)	101 (33.3)	
Hypertension	302 (68.3)	95 (68.3)	207 (68.3)	1.000
Hypercholesteraemia	341 (77.1)	101 (72.7)	240 (79.5)	0.142
Diabetes	103 (23.3)	53 (38.1)	50 (16.5)	0.000
BMI (kg/m^2^)/mean	28.6 ± 5.7	28.1 ± 6.0	28.8 ± 5.5	0.262
Examination and investigation				
LVEF (*n* = 434)				
- Good	323 (74.4)	101 (74.3)	222 (74.5)	0.167
- Fair	88 (20.3)	24 (17.6)	64 (21.5)	
- Poor	23 (5.3)	11 (8.1)	12 (4.0)	
Number diseased vessels (*n* = 435)				
- 0	79 (18.2)	28 (20.6)	51 (17.1)	0.799
- 1	34 (7.8)	10 (7.4)	24 (8.0)	
- 2	80 (18.4)	26 (19.1)	54 (18.1)	
- 3	242 (55.6)	72 (52.9)	170 (56.9)	
Pre-operative risk assessment				
EuroSCORE (median [IQR])	4 [1–5]	5 [2–6]	3 [1–4]	0.000
Intra-operative details				
Operative priority				
-Elective	308 (69.7)	73 (52.5)	235 (77.6)	0.000
Operation performed				
- CABG	298 (67.4)	86 (61.9)	212 (70.0)	0.103
- AVR	61 (13.8)	18 (12.9)	43 (14.2)	
- MVR	10 (2.3)	5 (3.6)	5 (1.7)	
- CABG + AVR	35 (7.9)	14 (10.1)	21 (6.9)	
- CABG + MVR	0 (0.0)	0 (0.0)	0 (0.0)	
- AVR + MVR	3 (0.7)	0 (0.0)	3 (1.0)	
- CABG + AVR + MVR	2 (0.5)	0 (0.0)	2 (0.7)	
- Other	33 (7.5)	16 (11.5)	17 (5.6)	
Duration of operation (min)	224.0 ± 54.1	230.3 ± 64.5	221.0 ± 48.3	0.098
Cardiopulmonary bypass used	410 (93.4)	139 (92.8)	281 (93.7)	0.837
Cardiopulmonary bypass time (*n* = 428)	79.0 ± 35.8	80.8 ± 41.6	78.2 ± 32.8	0.495
Aortic cross clamp time	51.0 ± 24.9	52.7 ± 29.5	50.26 ± 22.6	0.362
Outcome				
Number of hours ventilated (h) (*n* = 392)	9.84 ± 58.2	8.0 ± 6.6	10.6 ± 69.3	0.690
Length of ICU stay (mean/days) (*n* = 416)	2.0 ± 3.5	3.0 ± 5.7	1.6 ± 1.6	0.000
Readmitted to ICU	15 (3.4)	11 (8.4)	4 (1.4)	0.001
Return to theatre	21 (4.8)	13 (9.6)	8 (2.7)	0.003
Total length of hospital stay (mean/days)	11.8 ± 11.7	15.2 ± 17.2	10.2 ± 7.6	0.000

### Statistical analysis

All statistical analyses were performed in Stata version 13 (StataCorp Texas).

Baseline characteristics by anaemia were compared using Fisher’s exact test for categorical variables and Mann-Whitney *U* test for continuous data. The association of transfusion with anaemia after adjustment for covariates (age, gender and EuroSCORE) was assessed using a logistic regression model, and the odds ratio and 95% confidence interval were obtained. Associations with individual C-POMS morbidities were examined using random intercept logistic regression models. *p* values were corrected for multiple comparisons over the 13 morbidities using the Bonferroni correction.

Hb concentration at each time point was divided into quintiles, and differences in C-POMS score were tested between quintiles using Kruskal-Wallis test and a non-parametric test for trend across ordered groups (Cuzick 1985). Differences in C-POMS by quintile over all time points were estimated using a random intercept model with time fitted as a fixed effect.

Correlations of Hb with LOS and C-POMS score were assessed by Spearman rank correlation, and multivariate models were fitted for patient LOS and C-POMS score using ordinal logistic regression and random intercept models, respectively. Terms for both EuroSCORE and Hb were fitted as quintiles in the multivariate models as their distributions differed significantly from normality.

## Results

### Baseline characteristics

Of 748 potentially eligible patients undergoing cardiac surgery, 520 (69.5%) were screened (due to researcher availability) and 464 (89.2%) consented to participate. Fourteen participants subsequently became ineligible, leaving 450 who completed the study. Six participants declined for their data to be used outside the development of C-POMS, and a further two patients were without pre-operative Hb results, leaving 442 patients for analysis in this study.

Table 2 summarises the participants’ characteristics. Overall, the majority were White British (377, 86.1%) and male (350, 79.2%) with a mean age of 66.5 years (range 19 to 91 years). Seven patients (1.6%) were receiving renal dialysis while 50 (11.3%) had gastrointestinal disease. Most underwent isolated coronary artery bypass graft (CABG) surgery (298, 67.4%) and received cardiopulmonary bypass (410, 93.4%). Overall, the patients remained in the ICU and hospital for 2.0 and 11.8 days, respectively.

### Pre-operative anaemia

The overall median Hb was 135 (range 79 to 173). Pre-operative anaemia was present in 31.5% (139/442) participants. The median Hb in the anaemic group was 116 (range 79 to 129 g/l) and 140 (range 120 to 173 g/l) in the non-anaemic groups (*p* = 0.000).

Table 2 shows the comparison of the pre-, intra- and post-operative characteristics between those with and without anaemia. Patients with pre-operative anaemia were older (69.5 vs 65.1 years, *p* = 0.000), less likely to be of White British ethnicity (76.8 vs 90.3%, *p* = 0.001) and more likely to be receiving pre-operative dialysis (4.3 vs 0.3%, *p* = 0.005) than non-anaemic participants. Those with anaemia were also more likely to have a history of cerebrovascular accident (6.5 vs 2.3%, *p* = 0.05), gastrointestinal (GI) disease (16.5 vs 8.9%, *p* = 0.023) or congestive heart failure (CHF) (6.2 vs 1.7%, *p* = 0.034) and to be diabetic (38.1 vs 16.5%, *p* = 0.000). Thus, as would be expected, anaemic patients had a higher EuroSCORE (5 vs 3, *p* = 0.000) and were more likely to be undergoing urgent (non-elective) surgery (47.5 vs 22.4%, *p* = 0.000). Furthermore, compared to non-anaemic patients, those with anaemia were more likely to return to theatre (9.6 vs 2.7%, *p* = 0.003), be readmitted to the ICU (8.4 vs 1.4%, *p* = 0.001) and so to stay longer in the ICU (3.0 vs 1.6 days, *p* = 0.000) and in hospital (15.2 vs 10.2 days, *p* = 0.000).

### Pre-operative anaemia and RBC transfusion

Pre-operative anaemic patients were more likely to receive a RBC transfusion than non-anaemic patients (39.6 vs 14.5%, unadjusted odds ratio (OR) (95%CI) 3.85 (2.42–6.15) *p* < 0.0001) and, if transfused, to receive more units (2 vs 1 unit, *p* = 0.04). The association between anaemia and transfusion remained after adjustment for age, gender, EuroSCORE and LOS. Overall, anaemic patients had over three times the odds (OR 3.08, 95%CI 1.88–5.06, *p* < 0.001) of requiring a RBC transfusion than non-anaemic patients (14.1 vs 33.6%).

**Fig. 1:**
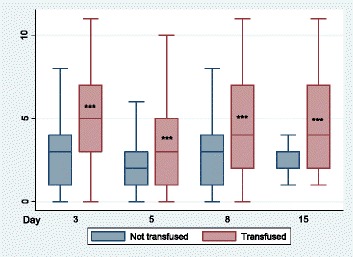
C-POMS summary score by red blood cell transfusion

Patients who received a RBC transfusion remained in hospital 5 days longer than those who did not (LOS 11 vs 6 days, *p* < 0.0001) and had a significantly higher C-POMS score on all days (D3 5 vs 3, D5 3 vs 2, D8 4 vs 3, D15 4 vs 2, all *p* < 0.001) (Fig. 1). Furthermore, RBC transfusion was associated with pulmonary, renal, GI, neurological, endocrine and ambulation morbidities (*p* 0.026 to <0.001), independent of Hb (Table 3).

**Table 3:** Morbidity outcome by domain following RBC transfusion

C-POMS morbidity domain	OR (95%CI)^a^	*p* value	Bonferroni adjusted *p* value
Pulmonary	3.55 (1.87–6.73)	0.0001	0.001
Infectious	1.71 (0.71–4.15)	0.232	1.00
Renal	4.52 (1.88–10.83)	0.001	0.013
Gastrointestinal	2.58 (1.42–4.68)	0.002	0.026
Cardiovascular	4.14 (1.37–12.47)	0.012	0.156
Neurological	5.89 (2.65–13.09)	0.00001	0.0001
Haematological	2.98 (1.20–7.41)	0.019	0.247
Wound	1.89 (0.66–5.40)	0.237	1.000
Pain	1.39 (0.51–3.78)	0.514	1.000
Endocrine	4.40 (1.97–9.84)	0.0003	0.004
Electrolyte	0.66 (0.14–3.08)	0.594	1.000
Review	1.97 (1.06–3.64)	0.032	0.416
Assisted ambulation	6.49 (2.57–16.40)	0.00008	0.001

^a^Odds ratio for association of transfusion with domain after adjustment for age, gender, time point and Hb

### Pre-operative anaemia and C-POMS score

**Fig. 2:**
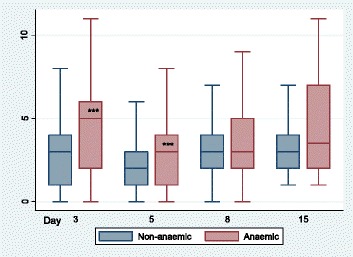
C-POMS summary score by pre-operative anaemia

Pre-operative Hb was correlated with C-POMS score on D3 (rho −0.28, *p* < 0.0001) and D5 (rho −0.18, *p* = 0.0002) but not D8 (rho −0.143, *p* = 0.06) or D15 (rho −0.28, *p* = 0.06). Patients with pre-operative anaemia had a significantly higher C-POMS score on D3 and D5 than non-anaemic patients (5 vs 3 and 3 vs 2, respectively, both *p* < 0.0001) but not on D8 (3 vs 3, *p* = 0.32) or D15 (3.5 vs 3, *p* = 0.27) (Fig. 2). Pre-operative anaemia was associated with renal (*p* < 0.001) and assisted ambulation (*p* = 0.003) but no other C-POMS domains (Table 4).

**Table 4:** Pre-operative anaemia and morbidity outcome by domain

C-POMS morbidity domain	OR (95%CI)^a^	*p* value	Bonferroni adjusted *p* value
Pulmonary	1.60 (0.93–2.76)	0.088	1.00
Infectious	1.87 (0.84–4.17)	0.125	1.00
Renal	6.88 (2.97–15.92)	0.000006	0.00008
Gastrointestinal	0.89 (0.52–1.53)	0.674	1.000
Cardiovascular	1.64 (0.62–4.31)	0.319	1.000
Neurological	0.81 (0.40–1.66)	0.571	1.000
Haematological	1.35 (0.60–3.03)	0.467	1.000
Wound	2.12 (0.80–5.62)	0.133	1.000
Pain	1.53 (0.63–3.75)	0.349	1.000
Endocrine	2.83 (1.38–5.80)	0.005	0.065
Electrolyte	2.73 (0.70–10.68)	0.150	1.000
Review	1.22 (0.68–2.20)	0.510	1.000
Assisted ambulation	4.96 (2.16–11.38)	0.0002	0.0026

^a^Odds ratio for association of anaemia with domain after adjustment for age, gender, time point and transfusion

Both pre-operative anaemia and transfusion requirement were independently associated with an increased C-POMS score (Table 5). Pre-operative anaemia was associated with a 0.55 (se 0.20) *p* = 0.005 increase in score, while RBC transfusion requirement was associated with an increase score of 1.23 (se 0.22) (*p* < 0.0001). If Hb replaced anaemia in this statistical model, both Hb and transfusion are independently associated with C-POMS score. C-POMS score decreases with every one quintile increase in Hb (B (se) −0.18 (0.06) *p* = 0.003) and increases by 1.19 with transfusion (se 0.22) (*p* < 0.0001). Increased age was also independently associated with increased C-POMS score in both models.

**Table 5:** Multivariate models for associations with C-POMS

		Model including anaemia		Model including Hb	
		*B* (se)	*p* value	*B* (se)	*p* value
Age	10-year increase	−0.15 (0.10)	0.13	−0.16 (0.10)	0.11
Gender	Female/male	−0.15 (0.22)	0.49	−0.34 (0.23)	0.13
EuroSCORE	Per quintile	0.48 (0.08)	<0.0001	0.48 (0.08)	<0.0001
Anaemia	Yes/no	0.55 (0.20)	0.005	–	–
Hb	1 SD increase	–	–	−0.29 (0.10)	0.003
Transfusion	Yes/no	1.23 (0.22)	<0.0001	1.19 (0.22)	<0.0001

### Pre-operative anaemia and hospital LOS

Pre-operative Hb was correlated with hospital LOS (rho −0.32, *p* < 0.00001). When compared to patients with Hb >14.6 (quintile 5), those with Hb <12.1 (quintile 1) had a higher morbidity score on D3 (5 vs 2, *p* < 0.0001) and D5 (3 vs 2, *p* = 0.007) and stayed in hospital for an additional 4 days (LOS 10 vs 6 days, *p* < 0.001) (Table 6). Pre-operative anaemic patients remained in hospital 2 days longer than non-anaemic patients (8 (inter-quartile range (IQR) 6–15) vs 6 (IQR 5–9) days, *p* < 0.0001, respectively).

**Table 6:** Median C-POMS score and hospital length of stay by quintile of Hb

Quintile	D3	D5	D8	D15	All time points	Hospital LOS
	Median [IQR]	Median [IQR]	Median [IQR]	Median [IQR]	*B* ^a^ (se)	Median [IQR]
1 (≤121)	5 [2–6] 90	3 [1–5] 87	4 [2–6] 54	4 [2–7.5] 20	0	10 [6–17.5] 88
2 (122–131)	4 [2–5] 93	2 [1–4] 90	3 [2–4] 43	3 [2–3] 7	−0.82 (0.29)	7.5 [6–11] 90
3 (132–138)	2 [1–5] 82	2 [1–3] 80	3 [1–4] 31	2 [2–6] 7	−1.34 (0.30)	6.5 [5–10] 82
4 (139–146)	3 [1–5] 89	2 [1–3] 82	3 [2–5] 29	3 [1–4] 6	−1.25 (0.29)	6 [5–9] 88
5 (>146)	2 [1–4] 85	2 [1–3] 78	3 [1–4] 19	2.5 [1.5–3] 4	−1.71 (0.29)	6 [5–8] 86
*p* value	<0.0001	0.007	0.28	0.67	<0.0001	<0.0001
*p* value (trend)	<0.0001	0.001	0.16	0.15	<0.0001	<0.0001

*IQR* inter-quartile range

^a^Difference in C-POMS score for each quintile compared to quintile 1 after adjustment for day of follow-up

Both pre-operative anaemia (OR 1.65, 95%CI 1.12–2.44, *p* = 0.01) and RBC transfusion requirement (OR 2.40, 95%CI 1.55–3.72, *p* < 0.001) were independently associated with increased hospital LOS (Table 7). If Hb level replaces pre-operative anaemia in the analysis model, there is a linear decrease in LOS over the five quintiles with odds ratios vs quintile 1 of 0.78, 0.64, 0.49 and 0.42 for quintiles 2 to 5. Lower Hb (OR per quintile of Hb 0.80, 95%CI 0.70–0.92, *p* = 0.001) and RBC transfusion requirement (OR 2.33, 95%CI 1.50–3.60, *p* = 0.0002) were independently associated with increased hospital LOS.

**Table 7:** Multivariate models for length of stay

	Model including anaemia			Model including Hb	
		OR (95%CI)	*p* value	OR (95%CI)	*p* value
Age	10-year increase	0.77 (0.64–0.94)	0.009	0.76 (0.63–0.92)	0.006
Gender	Female/male	1.24 (0.80–1.91)	0.34	1.01 (0.65–1.57)	0.95
EuroSCORE	Per quintile	1.85 (1.57–2.19)	<0.0001	1.83 (1.55–2.16)	<0.0001
Hb	Yes/no	–	–	0.71 (0.59–0.86)	0.001
Anaemia	1 SD increase	1.65 (1.12–2.44)	0.01	–	–
Transfusion	Yes/no	2.40 (1.55–3.72)	<0.0001	2.25 (1.45–3.49)	0.0003

## Discussion

Pre-operative anaemia has been associated with adverse outcome after cardiac surgery. However, whether anaemia is an independent risk factor for general morbidity after cardiac surgery (Fowler et al.) and the scale of this impact on total morbidity burden (TMB) has not previously been reported. Thus, our study explored the effect of pre-operative anaemia and RBC transfusion on total morbidity burden after cardiac surgery. Firstly, we found that compared to non-anaemic patients, pre-operative anaemic patients had significantly higher TMB (C-POMS scores) on D3 and D5, significantly more renal and ambulation morbidities and stayed in ICU and hospital an extra 1.4 days and an extra 2 days, respectively. As pre-operative anaemia was independently associated with increased TMB, reduction of post-operative morbidity might be achieved by treating pre-operative anaemia. Indeed, pre-operative optimization of anaemia in the UK is recommended (Service UKBT and T 2007; Department of Health 2007) as part of the patient blood management plan, with the use of intravenous (IV) iron if surgery may be delayed due to the time needed for oral iron to take effect (ERP Programme 2010). However, although IV iron therapy for anaemia has been shown to effectively treat anaemia in medical (Usmanov et al. 2008), and non-cardiac pre-operative settings (Munoz et al. 2009), the effect on cardiac surgical patients is not yet confirmed due to the low level of evidence available (Hogan et al. 2015). Thus, further prospective evidence in cardiac surgery patients is required before any recommendation for the use of IV iron to treat pre-operative anaemia in these patients can be made. Secondly, blood is a limited resource and is associated with high transfusion costs (Department of Health 2007), administration incidents and risks (Group SS 2014) and specifically poorer outcome in cardiac surgical patients (Galas et al. 2013). Our results found RBC transfusion to be independently associated with TMB, and patients spent an extra 5 days in hospital. Thus, strategies to reduce RBC use may reduce transfusion errors, reduce healthcare costs and improve patient well-being. However, although there are considerable differences in transfusion triggers across UK cardiac surgery centres (Murphy et al. 2013), restrictive transfusion protocols (Ternström et al. 2014) and patient blood management systems (Gross et al. 2015) do not appear to reduce post-operative morbidity in all instances, with the TITRe2 trial suggesting liberal transfusion may actually be superior after cardiac surgery (Murphy et al. 2015). This again raises the question on whether it is anaemia or RBC transfusion that carries the greatest risk (Vincent 2015; Du Pont-Thibodeau et al. 2014), and hence, exploring TMB in future anaemia and transfusion studies in cardiac surgery is needed. Adding further complexity to our understanding of pre-operative anaemia, treatment strategies and outcome, hepcidin, the principal regulator of systemic iron homeostasis, has been found to be an independent risk factor for poor outcome (Hung et al. 2015). This provides a new variable for consideration in further work, which is much needed before any conclusions can be made.

Where evidence exists, our study is comparable to other studies in terms of incidence of anaemia (De Santo et al. 2009; Kulier et al. 2007) and medical history (De Santo et al. 2009; Kulier et al. 2007). Our results were also consistent with others identifying pre-operative anaemia as a risk factor for post-operative renal complications (Cladellas et al.; Miceli et al. 2014; Fowler et al.) but not for cardiovascular complications (Cladellas et al.; Miceli et al. 2014; Fowler et al.; Carrascal et al. 2010). However, our findings did not suggest pre-operative anaemia to be associated with stroke (Miceli et al. 2014; Fowler et al.), infection (Cladellas et al.; Fowler et al.) or respiratory failure (Carrascal et al. 2010) as has been found previously. This is likely to be due to difference in definitions used between the studies, and thus the use of a standardised framework, like C-POMS, is advocated for future morbidity outcome after cardiac surgery studies.

There are four main limitations with our study. Firstly, pre-operative baseline characteristics obtained from the Society of Cardiothoracic Surgery of Great Britain and Ireland local database were 93.9% complete. It is possible the small amount of missing data may have had an influence on comparisons on the baseline characteristics. Secondly, although C-POMS is a validated tool for the description and quantification of morbidity after cardiac surgery (Sanders et al.), there are limitations to its use (Sanders et al.). This includes transient morbidities which may be missed on non-data collection days and that fluctuations cannot be tracked. Thirdly, as it is recommended that treatment of pre-operative anaemia should rely on the diagnosis of the type of anaemia, identifying the underlying cause or disease (Weiss and Goodnough 2005), we had intended to explore outcome by type of anaemia. However, since only 1.4% (2/139) of anaemic patients in our study had pre-operative haematinic profiles available, this was not feasible. Finally, we cannot prove that the associations we report are causal. Investigating this issue will require interventional studies to mitigate against pre-operative anaemia and post-operative transfusion, and we would advocate for such trials to take place.

## Conclusions

In conclusion, while previous evidence is inconclusive on the effect of pre-operative anaemia-specific morbidity outcome (for example stroke and renal dysfunction) after cardiac surgery, our study suggests that pre-operative anaemia and RBC transfusion use are independently associated with significant overall total morbidity burden following cardiac surgery. Thus, strategies to reduce pre-operative anaemia and RBC transfusion need are important. However, understanding that TMB (at the level of detail that the C-POMS tool permits) associated with pre-operative anaemia and RBC transfusion may assist in post-operative patient management to reduce morbidity, especially if it is not possible to ascertain whether it is anaemia or RBC transfusion that carries the greatest risk to patient well-being and recovery. We would recommend the use of the C-POMS tool as a standard morbidity outcome measurement tool in further studies to explore this and whether interventions implemented to reduce post-operative morbidity burden actually do reduce TMB as measured using the C-POMS tool.

## Abbreviations

ATP: Adenosine triphosphate
AVR: Aortic valve replacement
BMI: Body mass index
CABG: Coronary artery bypass graft
CHF: Congestive heart failure
C-POMS: Cardiac Post-Operative Morbidity Score
CRP: C-reactive protein
D3 (D5, D8, D15): Day 3 (day 5, day 8, day 15)
GI: Gastrointestinal
Hb: Haemoglobin
ICU: Intensive care unit
INR: International normalised ratio
IQR: Inter-quartile range
IV: Intravenous
LD: Leucocyte depletion
LOS: Length of stay
LVEF: Left ventricular ejection fraction
MI: Myocardial infarction
MVR: Mitral valve replacement
NYHA: New York Heart Association
OPA: Out-patient appointment
OR: Odds ratio
OT: Occupational therapy
RBC: Red blood cell
TMB: Total morbidity burden
UK: United Kingdom
